# Stem cell therapy for type 1 diabetes mellitus: a review of recent clinical trials

**DOI:** 10.1186/1758-5996-1-19

**Published:** 2009-10-16

**Authors:** Carlos Eduardo Barra Couri, Júlio César Voltarelli

**Affiliations:** 1 Department of Internal Medicine, School of Medicine of Ribeirão Preto, University of São Paulo, Ribeirão Preto, SP, Brazil

## Abstract

Stem cell therapy is one of the most promising treatments for the near future. It is expected that this kind of therapy can ameliorate or even reverse some diseases. With regard to type 1 diabetes, studies analyzing the therapeutic effects of stem cells in humans began in 2003 in the Hospital das Clínicas of the Faculty of Medicine of Ribeirão Preto - SP USP, Brazil, and since then other centers in different countries started to randomize patients in their clinical trials. Herein we summarize recent data about beta cell regeneration, different ways of immune intervention and what is being employed in type 1 diabetic patients with regard to stem cell repertoire to promote regeneration and/or preservation of beta cell mass.

The Diabetes Control and Complications Trial (DCCT) was a 7-year longitudinal study that demonstrated the importance of the intensive insulin therapy when compared to conventional treatment in the development of chronic complications in patients with type 1 diabetes mellitus (T1DM). This study also demonstrated another important issue: there is a reverse relationship between C-peptide levels (endogenous indicator of insulin secretion) chronic complications - that is, the higher the C-peptide levels, the lower the incidence of nephropathy, retinopathy and hypoglycemia. From such data, beta cell preservation has become an additional target in the management of T1DM [1].

## Immune interventions used in type 1 diabetes

Since the identification of the autoimmune etiology of T1DM in the late 1970s, the use of immunosuppressive agents began to occur. In 1981, Eliot and colleagues [2] treated newly diagnosed children with prednisone with the aim of stopping pancreatic β-cell destruction by the autoimmune process. Urinary C-peptide levels in the group treated with corticosteroid were significantly higher than control for 1 year after therapy was initiated. Subsequently, short-term studies were conducted using azathioprine [3], azathioprine plus prednisone [4], and cyclosporine [5] and demonstrated a slower decline (or even some improvement) in plasma C-peptide levels. In those studies, some patients experienced short periods (<1 year) during which they were free from insulin treatment (Table 1). The chronic toxicity of immunosuppression and the loss of the metabolic benefits after the withdrawal of the immunosuppressive agents limited the routine use of these therapies.

**Table 1:** Effect of different immunomodulatory therapies on insulin-free period in patients with newly diagnosed type 1 diabetes mellitus

**Immunomodulating therapy**	**Treatment length**	**Number of patients free from/total number of patients**	**Period free from insulin (average)**
Prednisone^2^	12 months (daily use)	4/17	3 months
Prednisone + azathioprine^4^	12 months (daily use)	10/20	1 week
Azathioprine^3^	12 months (daily use)	0/24	0
Ciclosporin^5^	24 months (daily use)	53/122	10 months
Antibody anti-CD3^6^	E.V. application 6 days in a row	0	0
Heat shock protein^7^	1 S.C. application in time 0, + 1 and + 6 months	0	0
Anti-thymocyte globulin^8^	E.V. Applications 4 days in a row	0	0
Glutamic acid decarboxylase^9^	2 subcutaneous applications with 1-month interval	0	0

Since 2000, studies have been published on acute immunomodulating therapies that are theoretically aimed at providing longer immunologic effect. These studies were performed with heat shock protein [6], anti-thymocyte globulin [6], antibody anti-CD3 [7] and glutamic acid decarboxylase (GAD) [9]. Such studies also showed several degrees of beta cell mass preservation; however, none of the patients became insulin-independent.

At the University of Florida, cell therapy for T1DM was conducted using autologous umbilical cord blood cells. Such cells have immunomodulating properties and are able to secrete cytokines that promote a decrease in the population of cytotoxic T lymphocytes and increase the population of regulatory T cells (T-regs), but they do not have proven regenerative properties. In this kind of therapy, a simple endovenous injection is applied and a study of this was recently conducted with a group of 21 patients who were an average of 5 years old and had had diabetes an average of 9 months, paired with a control group of patients receiving usual insulin therapy [10]. After a follow-up of 1 year, no significant biological differences were observed in the C-peptide levels and in the insulin doses used during that year ([10] and personal communication).

Another form of cell therapy is that performed by a group of researchers from Argentina, China and United States using stem cells from the patient's own bone marrow (including a conglomerate of mesenchymal stem cells and hematopoietic stem cells) obtained in a bone marrow biopsy. While still under anesthetic, these cells are infused by arterial catheterization directly into the patient's pancreas. This therapy was performed in 22 T1DM patients and in 31 type 2 diabetic patients. The authors did not publish complete data at all [11].

## Brazilian leadership in cell therapy for type 1 diabetes

By the end of 2003, cell therapy for T1DM started being performed in humans, and the world's first study was performed by the Divisions of Immunology and Endocrinology of the Hospital das Clínicas of the Faculty of Medicine of Ribeirão Preto - University of São Paulo - Brazil [12,13]. The basic inclusion criteria are age between 12 and 35 years and a diagnosis of T1DM less than 6 weeks prior to inclusion confirmed by positive serum levels of anti-GAD antibodies.

In the first stage of the protocol, called mobilization, a small dose of cyclophosphamide is administered intravenously to mobilize hematopoietic stem cells from the bone marrow to the peripheral blood. Afterwards, we apply daily subcutaneous injections of the granulocyte colony stimulating factor to proliferate circulating stem cells; these cells are then collected and frozen.

Ten to fifteen days later, we start the second phase, called the conditioning regimen, in which we use high dose immunosuppression with intravenous cyclophosphamide 200 mg/kg plus intravenous rabbit anti-thymocyte globulin 4.5 mg/kg with aim of 'turning off' the immune system, mainly the peripheral T cells that retain immunologic memory. Later, we intravenously re-infuse the previously collected hematopoietic stem cells, which do not have immunologic memory, to regenerate a new immune system that will not attack pancreatic beta cells. This procedure is called 'immunologic resetting' and although no beta cells are regenerated, those not yet destroyed are preserved. It is important to emphasize that evidence in the medical literature indicates that hematopoietic stem cells are unable to be differentiated into beta cells. This is why we include only newly diagnosed patients - that is, patients who still have residual beta cell mass to be preserved.

Using this procedure, we may maintain or increase the secretion of endogenous insulin, improving the metabolic control and obviously the risk of chronic complications. We emphasize that if we obtain increased C-peptide levels for long periods and keep good glycemic control, we may offer patients a lower risk of chronic complications from diabetes, even without suspending insulin therapy.

Up to December 2008, we have performed this procedure in 23 patients: 17 men and 6 women aged 13 to 31 with an average body mass index of 19.7 kg/m^2^, an average glycemia level of 395.6 mg/dl and an average glycosylated hemoglobin level of 8.4% at the time of diagnosis, and undergoing anti-GAD treatment ranging from 1.1 to 102 U/ml and using an average dose of 0.4 UI/kg/day soon before starting the treatment.

All patients are recommended to be on a diet and to exercise regularly within a regimen of intensive insulin therapy and carbohydrate counting to try and achieve the following goals: pre-prandial glycemia <120 mg/dL, postprandial glycemia <140 mg/dL and glycosylated hemoglobin (HbA1c) <7%.

Patients receive psychological guidance to understand that they still have T1DM and that they must maintain a routine of automonitoring of capillary glycemia and carbohydrate counting to refrain from food abuse. Compliance with these guidelines is a challenge for the multidisciplinary team.

Of the 23 patients included, 20 remained free from insulin for some period. Of these, 12 have been continuously insulin-free since treatment, 8 became transiently insulin-free and 3 maintained daily insulin doses. In the group of continuously insulin-free patients, most stopped daily insulin injections soon after stem cell infusion and the mean period free from insulin is 31 months (ranging from 14 to 52 months). There was a significant reduction in HbA1c compared with pretreatment values, with all measures below 7% during follow-up. In parallel, this group of patients had important increases in mean peak-stimulated C-peptide levels (0.8 nmol/L pre-treatment versus 2.9 nmol/L after 3 years; *P *< 0.05). Of note, all patients reported important improvement in their quality of life; complete data will be published soon. Moreover, this long period without exogenous insulin use associated with progressive increases in C-peptide levels in multiple patients practically rules out the hypothesis of a prolonged honeymoon phase in this group.

Eight patients became transiently insulin-free for periods of 6 to 47 months (mean 17.7 months) However, they have excellent glucose control using small doses of insulin. Interestingly, in two patients who relapsed after being insulin free for 43 and 47 months, respectively, we opted to add sitagliptin (a dipeptidyl peptidase-4 inhibitor) 100 mg per day orally 4 and 2 months after resumption of insulin treatment. Both became insulin-free again 1 and 2 months later, and have remained free from exogenous insulin for more than 5 and 6 months, respectively. Peak-stimulated C-peptide levels declined in both patients before insulin treatment was resumed; however, C-peptide levels increased again after sitagliptin prescription, corresponding with the period of insulin-independence. Aside from this, we believe that suppression of glucagon levels can also explain the rapid insulin suspension after sitagliptin use. Additionally, immunomodulating effects of sitagliptin are speculated. So, this is the first report of complete insulin suspension in individuals with T1DM using sitagliptin [14].

In the whole group of eight patients who were transiently insulin-free, there was a significant increase in C-peptide levels from 0.6 nmol/L pre-treatment to 1.7 nmol/L 4 years after treatment (*P *< 0.05). To the best of our knowledge, this is the most prolonged period of C-peptide increase (up to 4 years) in interventional trials aiming at beta cell preservation.

Only three patients did not experience any period free from insulin. One presented with diabetic ketoacidosis at diagnosis and received glucocorticoids to prevent rabbit anti-thymocyte globulin reactions, one developed diabetic ketoacidosis before enrollment, and one had inadvertently received steroids (300 mg hydrocortisone) along with stem cell infusion. None of them achieved HbA1c levels less than 7% in spite of progressive increase in daily insulin doses (>0.8 IU/kg/day).

The majority of the adverse effects related to treatment were mild, including nausea, vomiting, fever, hyporexia and alopecia. With regard to severe adverse effects, two patients presented bilateral nosocomial pneumonia that completely responded to intravenous broad spectrum antibiotics; there was no mortality. During long-term follow-up, there was one case each of Graves' disease, transient hypergonadotropic hypogonadism, and autoimmune hypothyroidism. Nine patients had post-transplant hypospermia. Two patients fathered children 2 years after transplant. There was no mortality.

## Expansion of global research centers

In 2006, a Chinese group from the University of Naijing started a protocol similar to the one in Ribeirão Preto, with the difference that the stem cell infusion was 50% into the peripheral vein and 50% straight into the pancreas through arterial catheterization. Of the five patients initially treated less than 3 months after diagnosis, four became insulin-independent (two of them only transiently) and one had a 50% decrease in insulin dose. All 11 patients who had been diagnosed over 3 months before treatment kept using insulin.

In August 2008, in Chicago, we started an international multicentric randomized research protocol project, similar to that performed in Brazil, to test the effect of 'immunologic resetting' more widely. This study is being evaluated by the US Food and Drug Administration and by American research ethics organizations and will commence in 2009 with the collaboration of our team.

## Perspectives

Despite the encouraging results obtained so far, we realize the limitations of our study, such as risk of infections, elevated cost, need of an ultra-specialized team, requirement for patients to have been diagnosed no later than 6 weeks prior to treatment, and so on.

Our next step, the use of mesenchymal stem cells, has already been performed. Therapy with mesenchymal stem cells plays a very attractive theoretical role: regeneration of beta cells and immunomodulating action. Thus, its use in humans would eliminate the use of chemotherapeutic agents. Studies *in vitro *using growth factors could differentiate mesenchymal cells and beta cells. These cells in animal models of T1DM have been able to reverse hyperglycemia. Stem cells are the largest focus of interest among diabetologists working with cell therapy around the world and were a matter of wide debate in the 2008 American Congress of Diabetes in San Francisco.

In 2008, our group of researchers at the Medical School of Ribeirao Preto - USP started pioneering studies in humans with T1DM using mesenchymal stem cells. The protocol includes bone marrow biopsy under general anesthesia in first-degree relatives for the collection of mesenchymal cells. These cells are sent to a laboratory to be stimulated to proliferate for a month and are later infused into the patient through a gelatinous solution of approximately 100 ml; there is no need for chemotherapy. The patient is hospitalized for 1 day but only as a precaution. After 1 month, the patient receives another infusion. So far, we are not yet sure how many infusions will be necessary. Inclusion criteria are age 12 to 35 years, diagnosis of T1DM less than 4 weeks prior to treatment without ketoacidosis and positive serum levels of anti-GAD. So far, two patients have been included in this protocol and, as soon as we have a proper follow-up, the results will be published.

## Conclusion

Cell therapy is one of the research fields that have been widely expanding in the past few years. There is much expectation around this field, especially with regard to diabetes mellitus as it is a chronic disease from which organic and psychosocial data could be derived. We are not sure if we will obtain a 'cure', but we are glad to know that Brazilian science is collaborating decisively in the search for revelatory results.

## Abbreviations

GAD: glutamic acid decarboxylase; HbA1c: glycosylated hemoglobin; T1DM: type 1 diabetes mellitus.

## Competing interests

The authors declare that they have no competing interests.

## Authors' contributions

All the authors and participated in the manuscript design and drafting. All authors participated in study coordination and acquisition and interpretation of the data showed. All authors read and approved the final manuscript.
